# rs4246215 is targeted by hsa-miR1236 to regulate *FEN1* expression but is not associated with Fuchs’ endothelial corneal dystrophy

**DOI:** 10.1371/journal.pone.0204278

**Published:** 2018-09-27

**Authors:** Gargi Gouranga Nanda, Malloji Vinay Kumar, Laxmipriya Pradhan, Biswajit Padhy, Satabdi Sundaray, Sujata Das, Debasmita Pankaj Alone

**Affiliations:** 1 School of Biological Sciences, National Institute of Science Education and Research (NISER) Bhubaneswar, HBNI, P.O. Bhimpur-Padanpur, Jatni, Khurda, Odisha, India; 2 L. V. Prasad Eye Institute, SMTC Campus, Bhubaneswar, Odisha, India; Wayne State University, UNITED STATES

## Abstract

Fuchs’ Endothelial Corneal Dystrophy (FECD) is a genetically complex disorder that affects individuals above 40 years of age; molecular pathogenesis of its associated genes is poorly understood. This study aims at assessing the association of flap endonuclease 1 (*FEN1*) polymorphisms, c.-69G>A (rs174538) and c.4150G>T (rs4246215) with FECD. Comet assay analysis reaffirmed that endogenous DNA damage was greater in FECD individuals. However, genetic analysis in 79 FECD patients and 234 unrelated control individuals prove that both the *FEN1* polymorphisms, c.-69G>A (rs174538) and c.4150G>T (rs4246215), failed to show any genetic association with the FECD disease phenotype. *In silico* analysis and luciferase reporter assay identified ‘G’ allele of the 3’UTR located *FEN1* polymorphism c.4150G>T as the target for binding of hsa-miR-1236-3p. This study indicates that although *FEN1* polymorphisms, c.-69G>A (rs174538) and c.4150G>T (rs4246215) are not genetically associated with FECD, its transcript regulation reported in other diseases such as lung cancer which are genetically associated by rs4246215 could be mediated through miRNA, hsa-miR-1236-3p.

## Introduction

Fuchs’ endothelial corneal dystrophy (FECD) is an autosomal dominant, bilateral, age related disorder, known since 1910 after its published detailed description by Ernst Fuchs.[1] Disease manifestation involves decreased endothelial cell density and edematous cornea, which progressively consequent into decreased visual acuity. FECD is a genetically complex disorder with an increased predilection for females (2.5:1) and affects 3.9% of individuals over forty years of age in USA, from which it derives the name late-onset FECD.[2–4] On the contrary, a rarer variant of this disease known as early-onset FECD is also detected in individuals at their first decade and harboring autosomal dominant mutations in *COL8A2* gene.[5] Corneal transplantation is considered as the sole alternative for restoring vision in FECD affected individuals.[6] Over the past few years only handful of studies on FECD have been carried out in India that reported about 10.8% (113/1048) of total Endothelial Keratoplasty performed over a span of five-six years are due to FECD cases solely;[7, 8] indicating a prominent contribution of FECD in the total endothelial dystrophy cases in our country.

Linkage analysis and genome wide association studies have identified a plethora of genes and their genetic variations as risk factors associated with late-onset FECD (LO-FECD).[9–15] One of the disease pathomechanism involves increased oxidative stress and resultant apoptosis of corneal endothelial cells.[16, 17] In a recent study, one of the two polymorphisms, c.-69G>A (rs174538) and c.4150G>T (rs4246215), studied in Flap endonuclease 1 (*FEN1*) gene was genetically associated with LO-FECD in Polish population.[18] As a genome stabilization factor, *FEN1* is one of the component in DNA damage repair mechanism during long-patch base-excision repair (BER).[19] It has been established through previous studies that changes in *FEN1* gene expression can increase the susceptibility of DNA damage during oxidative injury.[20, 21] The current study aims at assessing the genetic association of the two *FEN1* polymorphisms 69G>A and c.4150G>T in Indian population. It also aims at investigating the functional role of the associated variant by evaluating its involvement in DNA damage and having a regulatory role as a miRNA target site.

## Materials and methods

### Participants and genetic analysis

**Fig 1:**
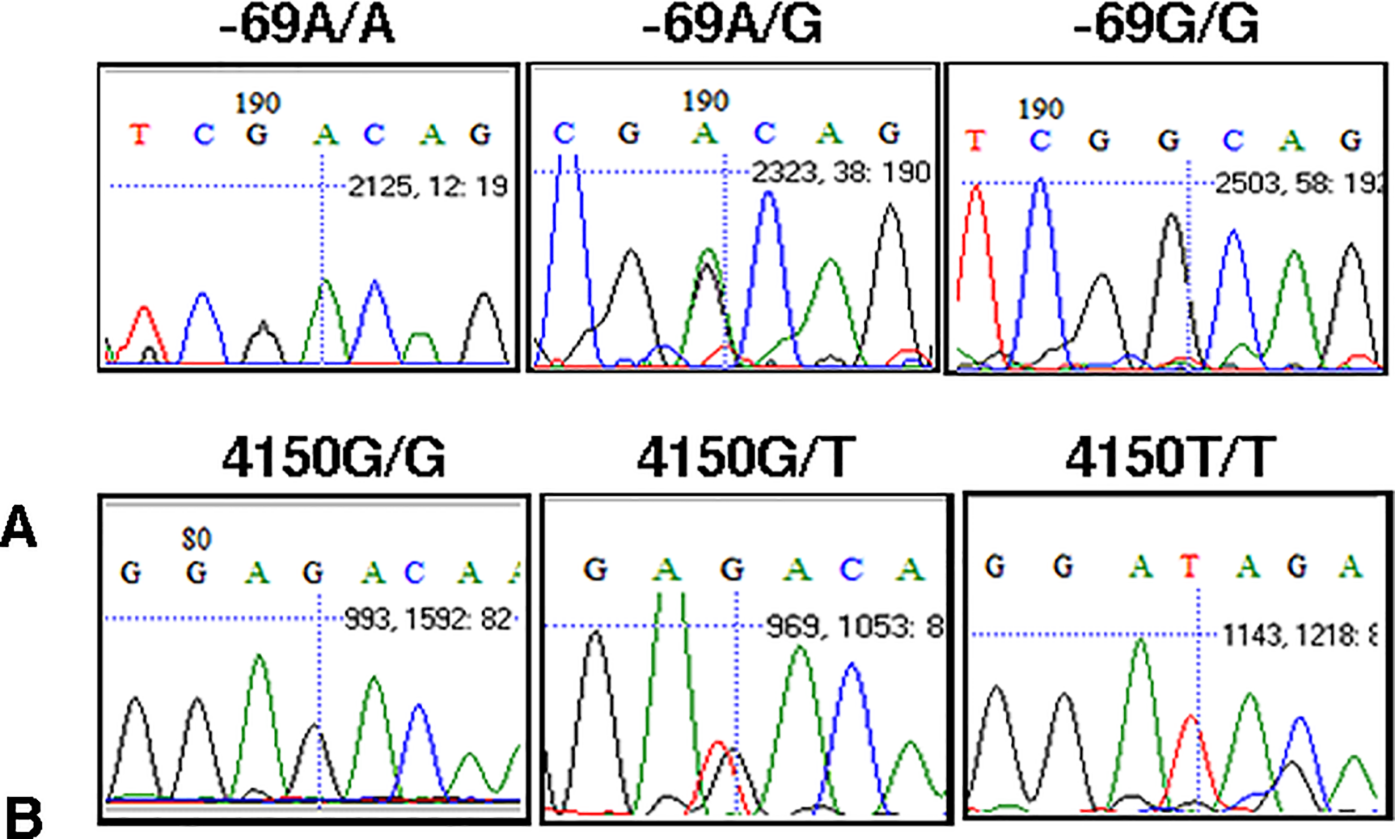
Representative sequence chromatographs of *FEN1* polymorphisms (A) c.-69G>A and B) c.4150G>T.

A population of 234 controls and 79 patients, of Indian origin, were recruited at L. V. Prasad Eye Institute (LVPEI) at Bhubaneswar, India after acquiring written consent from the participants. This study was approved by the Ethics review board from Institutional Ethics Committee (IEC) and Institutional Biosafety Committee (IBSC), National Institute of Science Education and Research (NISER) and Institutional Ethics Committee (IEC), LV Prasad Eye Institute (LVPEI). Recruitment of study participants was done as per the previously published report [22] and in accordance with the Declaration of Helsinki. Genomic DNA extracted from peripheral blood leucocytes of these individuals was used to genotype *FEN1* polymorphisms, rs174538 using 5’ CTCTCGCCCTTAGAAATCGC 3’ and 5’ GGCAACCAGTCCCTCCAG 3’ and rs4246215 using 5’ TATGTCAGGCTCAAACCAC 3’ and 5’ CAGCCAGTAATCAGTCACAA 3’ by Sanger sequencing (Fig 1).[22]

#### Comet assay

Endogenous DNA damage of the peripheral blood mononuclear cell (PBMC) samples was quantified by performing alkaline comet assay with the frozen blood leucocytes from the participants following previously published literature [23] and expressed as the natural log of Olive tail moment (Ln-OTM) of the comets after analysing individual fluorescent comet images on CASPLab v1.2.3beta2 comet analysis software (University of Wroclaw, Institute of Theoretical Physics, Poland).

### *In silico* prediction of miRNA sites in 3’UTR of *FEN1*

In order to analyse the putative miRNA binding sites at the 3’UTR of *FEN1*, the most frequently used computational algorithms such as miRWalk 2.0 (http://zmf.umm.uni-heidelberg.de/apps/zmf/mirwalk2/index.html), miRanda (http://www.microrna.org/microrna/home.do); TargetScan (http://www.targetscan.org); PicTar (http://www.pictar.org), RNA22 (https://cm.jefferson.edu/rna22/)32, FindTar3 (http://bio.sz.tsinghua.edu.cn) and SegalLab (https://genie.weizmann.ac.il/index.html) and SNPinfo database (http://snpinfo.niehs.nih.gov/) were used.

### Luciferase reporter assay

Target site for miRNA binding was assessed *in vitro* by reporter assays using pMIR-report luciferase vector (Invitrogen) containing CMV promoter and pGL4.74 Renilla vector as reporter control plasmid. Target oligos (67bp) for mIR-1236 as identified through bioinformatics analysis on the 3’UTR of *FEN1* gene (Table 1), were synthesized and annealed in 1X annealing buffer (30mM HEPES pH7.4, 100mM potassium acetate and 2mM magnesium acetate) at 90°C for 5 mins, followed by an hour incubation at 37°C. The annealed oligos were cloned into XhoI-NotI double digested pMIR-report vector at 3’UTR of the luciferase gene. The resultant pMIR-1236 clones (1μg) along with hsa-miR-1236-3p mimic (10pmoles, Invitrogen) and pGL4.74 vector (5ng) were transiently co-transfected into HEK293 at 80% confluency using Lipofectamine 2000 (Invitrogen). 24hr post-transfection, cells were harvested and the reporter activity of the transfected cells were measured using Dual-luciferase reporter assay system (Promega). Reporter activity for each assay group were measured in Varioskan Flash spectral scanning multimode reader (Thermo Scientific, USA). After normalization with Renilla reporter activity as transfection control, values obtained for each group were plotted as percent relative luciferase activity in comparison to empty pMIR vector.

**Table 1:** Oligomers designed for miRNA target analysis. For cloning into pMIR-report vector, these oligomers include restriction enzyme overhangs (denoted in lowercase), miRNA target site (in ***BOLD***) and position of c.4150G>T SNP (**G/T**).

Oligo name	Sequence (5' - 3')
WT1236/G	tcgagTGAAAGTGATAGATAGCAACAAGTTTTGGA***GAAGAGA***GAGGGAGATAAAAGGGGGA**G**ACAgc
WT1236/T	tcgagTGAAAGTGATAGATAGCAACAAGTTTTGGA***GAAGAGA***GAGGGAGATAAAAGGGGGA**T**ACAgc
MT1236/G	tcgagTGAAAGTGATAGATAGCAACAAGTTTTGGA***TTTTTTT***GAGGGAGATAAAAGGGGGA**G**ACAgc

### Relative transcript expression analysis

RNA from peripheral blood leucocytes was extracted (Nucleospin RNA blood kit, Mackerey-Nagel) and converted to cDNA using 3:1 (v/v) random hexamers and anchored oligo (dT) reverse transcription primers (Verso cDNA conversion kit, ThermoFisher Scientific). Expression of *FEN1* transcript in control and FECD samples was analyzed in triplicates, taking GAPDH expression as internal control, on ABI 7500 Real time-PCR system, using SYBR-green method. Primers used for qRT-PCR are, *FEN1* 5’ GGAGAGCGAGCTTAGGACCG 3’ and 5’CAACACAGAGGAGGGATGACTGG 3’ and *GAPDH* 5’ GAAGTCAGGTGGAGCGAGG 3’ and 5’ GCCCAATACGACCAAATCAGAG 3’. Transcript expression of *FEN1* in c.4150TT homozygotes was compared against c.4150GG individuals.

### Statistical analysis

To estimate the genetic power for the enrolled cases and control groups post hoc, G*Power v3.1.9.2 statistical power analysis software (University of Düsseldorf, Germany) was used. With the effect size of 0.5 (intermediate effect) and alpha error at 0.05, the genetic power of the sample size was estimated to be at 90.73%. The difference in age and gender distribution across cases and controls were computed by carrying out Student t- test and Fisher’s exact test respectively.

SPSS 23.0 statistical software for MacOS (IBM SPSS, Inc., Chicago, IL, USA) was used for assessing the genotyped polymorphisms for their association with FECD by employing Chi-squared test and logistic regression with age, gender and endothelial counts as covariates. Genotypic variables were coded as 0, 1 and 2 where the homozygous risk alleles in each case were weighed as 2. Haplotype frequency and association analysis, linkage disequilibrium (LD) estimation, LD plot generation and tests for deviance from Hardy Weinberg Equilibrium (HWE) was performed on Haploview4.2 (Broad Institute, Cambridge, MA, USA). Association of polymorphisms below the threshold of 5% was considered significant for this study.

Unpaired T-test with Welch’s correction was performed to analyse the differences between the LnOTM values of comets from each sample types and differences in normalized *FEN1* expression fold changes between the genotypes.

## Results

### FECD samples show significant DNA damage

**Fig 2:**
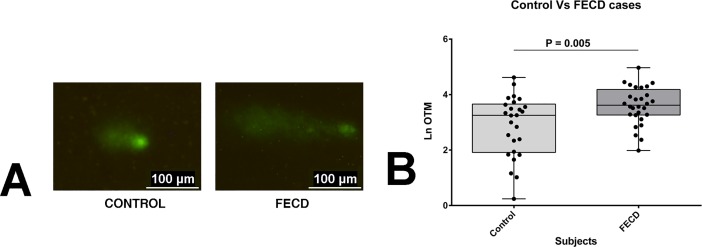
FECD lucocytes show increased endogenous DNA damage. (A) Representative images of comets from control and FECD peripheral leucocytes are shown. (B) Ln-OTM values of the comets are plotted to compare the DNA damage in FECD cases (n = 28) against controls (n = 24). Error bars indicate standard error (SE) in both directions.

Whole blood from 28 FECD patients and 24 individuals with healthy cornea were selected for comet assay considering the limited supply of post-operative corneal endothelial tissues from the study participants and genomic homogeneity between PBMCs and corneal cells in accordance with previously reported studies.[23, 24] Representative images of the comets segregated on the basis of affected status can be seen in Fig 2A. Endogenous DNA damage, expressed as Ln-OTM, was significantly higher in FECD cases (*P* value = 0.005, n = 28) as compared to their unrelated controls (n = 24, Fig 2B).

### *FEN1* polymorphisms are not associated with FECD risk in Indian cohort

Based on the comet data and previously reported genetic association study of *FEN1* with FECD in Polish population, we aimed to assess the genetic involvement of *FEN1* gene in conferring insufficient DNA repair and genotyped two polymorphisms present in its regulatory untranslated regions, c.-69G>A (rs174538; 5’UTR) and c.4150G>T (rs4246215; 3’UTR) in 79 FECD cases and 234 control individuals in Indian population. About 68% of the cases were female populated; thereby substantiating the fact that FECD is a female-biased disease reported worldwide. However, only 43% females were present in the control group. This generated statistically significant difference in gender distribution between the two groups (*P* value = 0.001). For genetic association tests, age-gender corrected samples were used. The mean age of the cumulative study population is 63.2 years. Considering the essential age-sensitive bracket for FECD ranging from 40–85 years, individuals ranging from 43–80 years for patients and 42–85 years for control groups have been included in this study to keep the age distribution unbiased (*P* value = 0.09; Table 2). Mean endothelial cell count was significantly low in FECD patients (1476 ± 284.3, *P* value<0.001) suggesting that these individuals suffer from progressive stage of endotheliopathy.

**Table 2:** Demographics and clinical characteristics of the study population.

Variables	Cases	Control	*P* Value
Number of Individuals	79	234	
Number of females (%)	54 (68)	101 (43)	0.001
Mean Age in years (SD)	61.08 (9.73)	63.32 (7.82)	0.09
Endothelial Count Mean (SD)	1476 (284.3)	2301 (349.1)	< 0.0001

Distribution of alleles in both the subject groups were under Hardy Weinberg equilibrium (HWE). Genotypic and allelic frequencies for *FEN1* c.-69G>A and *FEN1* c.4150G>T are as detailed in Table 3. With a genetic power of the current study at 90%, both the polymorphisms failed to project a genetic association with FECD. Neither of the minor alleles (rs174538: A, rs4246215: T) differed significantly between the two groups. Various association tests for the model of inheritance like dominant, recessive, and additive also confirmed the same; thereby eliminating *FEN1* polymorphisms, c.-69G>A and c.4150G>T as genetically associated with FECD.

**Table 3:** Genetic association of polymorphisms in *FEN1* gene with FECD. Allelic and genotypic distribution of SNPs, rs174538 and rs4246215 across patients and controls are tabulated. Polymorphism rs4246215 shows recessive mode of inheritance with the disease.

SNP	Type	Control%	FECD %	Tests	*P* Value	OR (95%CI)
rs174538	GG	69.2	68	ADD	0.68	0.99 (0.55–1.79)
(c.-69G>A)	AG	30.2	30	DOM	1	0.96 (0.53–1.73)
	AA	0.5	1	REC	0.46	2.5 (0.15–40.73)
				GENO	0.60	
Major Allele	G	83.3	84.8	ALLELIC	0.79	0.89 (0.52–1.50)
Minor Allele	A	16.7	15.2	PERM 10K	0.87	
rs4246215	GG	71	72	ADD	0.41	0.95 (0.52–1.71)
(c.4150G>T)	GT	24	25	DOM	0.54	1.71 (0.36–8.01)
	TT	4	2	REC	0.27	0.95 (0.52–1.71)
				GENO	0.29	
Major Allele	G	84.9	82.2	ALLELIC	0.48	1.22 (0.72–2.07)
Minor Allele	T	15.1	17.9	PERM 10K	0.67	

ADD: Additive, DOM: Dominant, REC: Recessive, GENO: Genotypic, PERM 10K: 10,000 permutation test, OR: Odds Ratio, CI: Confidence interval.

### *FEN1* polymorphism c.4150T acts as a target for hsa-miR-1236-3p

Although *FEN1* is not genetically associated with FECD, we simultaneously explored the possibility of any functional trait conferred by its 3’UTR variant, rs4246215 by scanning for putative miRNA binding sites on or around this SNP using computational algorithms like miRWalk, miRanda, TargetScan, PicTar2, RNA22, FindTar, and Segal Lab based on their alignment, energy, and mirsvr scores (Table 4). We identified about 274 miRNAs from TargetScan and nine miRNAs from MiRanda from the initial screening. Out of these, eight candidates were common between minimum of five algorithm outputs and only hsa-miR-1236-3p spans the SNP of interest. The binding site for hsa-miR-1236-3p includes ‘G’ allele from the polymorphic site and a seed region, GAAGAGA situated 19 bases upstream of it.

**Table 4:** Predicted miRNA targets on the 3’UTR of *FEN1* gene. EntrezID: 2237, RefseqID: NM_004111.

miRNA	MIMATid	miRWalk	miRanda	Pictar2	RNA22	Target-scan	FindTar3	Segal Lab	SNPinfo
hsa-miR-532-3p	MIMAT0004780	1	1	0	1	1	1	1	
hsa-miR-515-5p	MIMAT0002826	1	1	0	1	1	1	1	
hsa-miR-548k	MIMAT0005882	1	1	0	1	1		1	
hsa-miR-519e-5p	MIMAT0002828	1	1	0	1	1	1		
hsa-miR-2116-3p	MIMAT0011161	1	1	0	1	1	1		
hsa-miR-610	MIMAT0003278	1	1	0	1	1		1	
hsa-miR-4297	MIMAT0016846	1	1	0	1	1	1		
hsa-miR-942-5p	MIMAT0004985	1	1	0	1	1	1		
hsa-miR-1236-3p	MIMAT0005591		1			1			1

**Fig 3:**
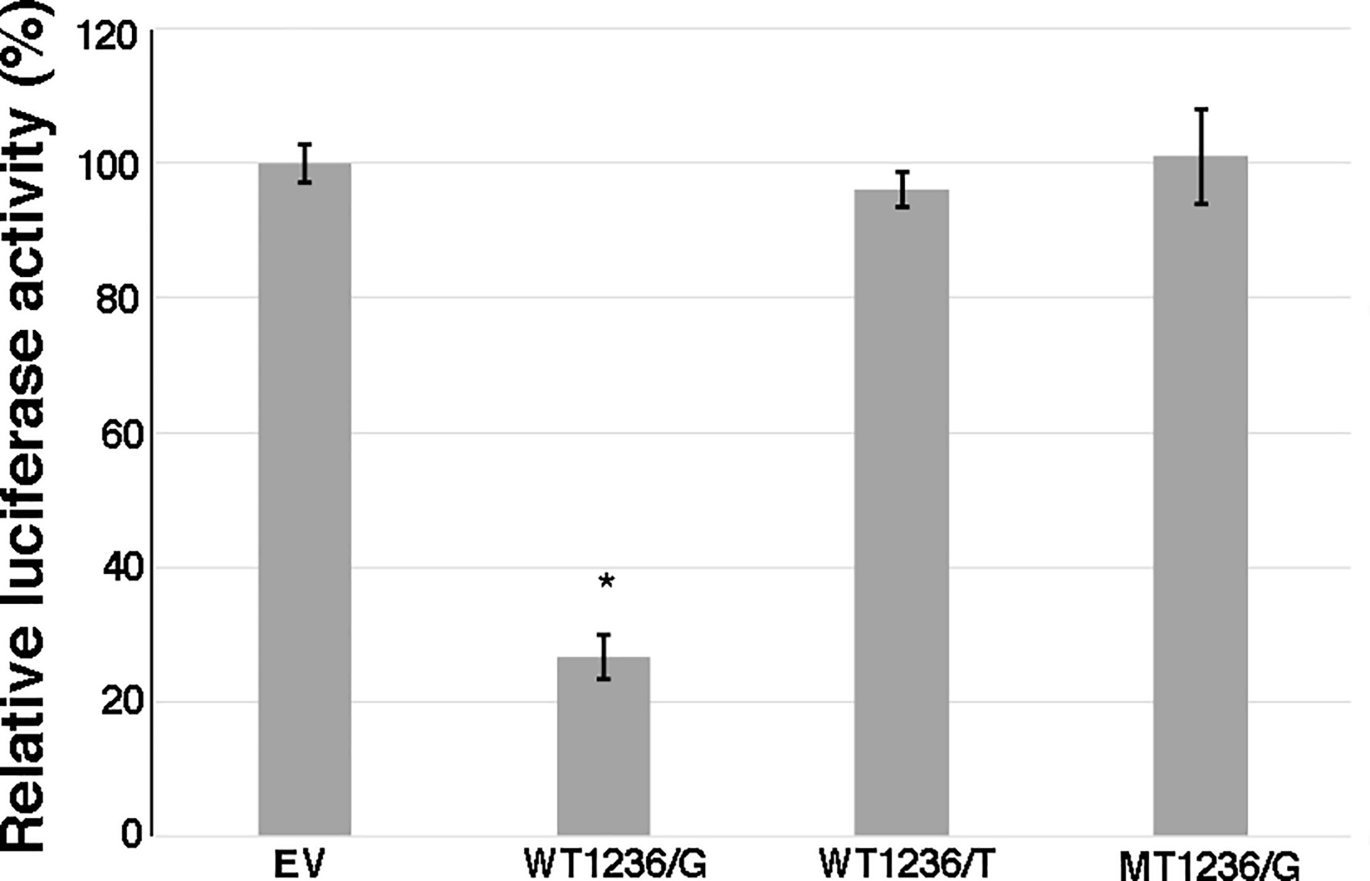
miRNA mediated reporter activity on 3’UTR regions of *FEN1* gene. Normalized luciferase activity in HEK293 cells transfected with hsa-miR-1236-3p mimic is shown to demonstrate the effect of allelic changes on luciferase activity. Trasnfected cells carrying contructs with ‘G’ allele exhibit reduced luciferase activity (26.6 ± 3.2) in presence of hsa-miR1236-3p as compared to those with ‘T’ allele (96.1 ± 2.5) or scrambled hsa-miR-1236-3p binding sequence with G allele at SNP position (MT1236/G; 101 ± 6.9). Luciferase acticity of cells transfected with empty pMIR-report vector (EV) were taken as control (100 ± 2.88). Error bars indicate standard error and * indicate *P* value = 0.008.

To validate this finding, we performed luciferase assay by transfecting HEK293 cells with constructs comprising of 60bp region from 3’UTR of *FEN1* gene, harboring target sites for hsa-miR1236-3p. Each of the three types of pMIR-constructs, WT1236/G, WT1236/T and MT1236/G, were individually co-transfected along with excess of hsa-miR1236-3p mimic into HEK293 cells. Regulatory effect was assessed by measuring the percent-luciferase activity in these cells. In presence of ‘G’ allele, we observed a significantly reduced reporter activity (26.6%, P = 0.008) than the cells with ‘T’ allele or mutant constructs. This suggests that both the miRNA target sequence (GAAGAGA) and ‘G’ allele at c.4150G>T polymorphic site is required for hsa-miR-1236-3p mediated downregulation of luciferase activity (Fig 3). This result indicates that rs4246215 can act as a functional variant when associated with a disease by regulating the expression *FEN1* gene.

**Fig 4:**
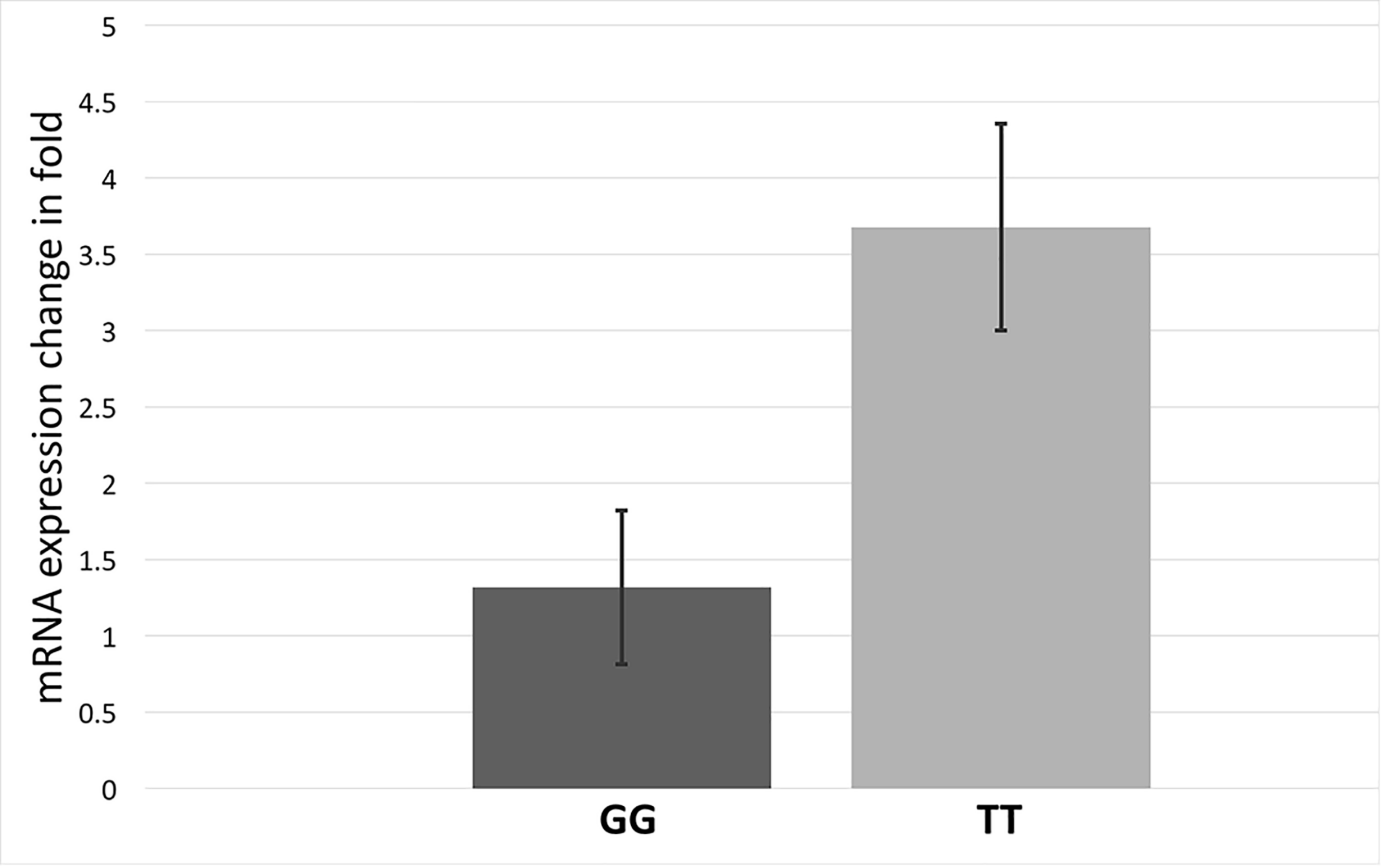
*FEN1* transcript expression in PBMCs of c.4150TT homozygotes was 2.5-fold upregulated than c.4150GG individuals.

Allele specific *FEN1* regulation was further validated through qRT-PCR with comparison between genotype specific blood samples. We observed a 2.5-fold over-expression of *FEN1* in c.4150TT homozygotes when compared with c.4150GG genotype individuals (Fig 4). This corroborates with the luciferase data and indicates that miRNA binding near rs4246215 suppresses the *FEN1* expression.

## Discussion

The current study highlights two important features: (1) it accurately negates the previous study that associates *FEN1* with FECD and finds experimental error as a reason for this discrepancy and (2) discovers a novel miRNA candidate that targets the 3’UTR of *FEN1* gene. Our comet assay analysis on peripheral leucocytes reaffirmed that FECD individuals have high endogenous DNA damage than controls; however, reported polymorphisms of *FEN1* may not be genetically involved for this phenotype. In contrast to the erroneous Polish report, both of the *FEN1* polymorphisms, c.-69G>A and c.4150G>T, did not show association with FECD in Indian population. Further analysis of these polymorphisms revealed a putative miRNA binding site on the 3’UTR, which could regulate its expression. Deregulated *FEN1* expression was associated with rs4246215 in lung cancer tissues [23]. This study indicates that although *FEN1* polymorphisms, c.-69G>A (rs174538) and c.4150G>T (rs4246215) are not genetically associated with FECD, its transcript regulation in FECD and other diseases such as lung cancer could be mediated through miRNA.

Upon performing a BLAST analysis on the primer pair utilized in the Polish study, it revealed that in addition to the intended 3’UTR of *FEN1*, an unintended region of *FEN1* pseudogene on chromosome 1 which shares 98% sequence homology with the *FEN1* gene on chromosome 11 would amplify. This error was confirmed when RFLP and Sanger sequencing of the same sample produced different genotypes. This makes the Polish data grossly erroneous and should be interpreted with caution. Our re-designed primer pair for rs4246215 correctly amplified this region and confirmed its non-association with FECD in Indian population.

The Polish report by Wojcik et al. 2014, was the first to report a genetic association of *FEN1* polymorphisms with FECD. Although the current study identified that their results might be erroneous, but it cannot disprove the possibility that *FEN1* polymorphisms may be associated with FECD in Polish population. As elaborated by Myles et al., disease-associated SNPs differ between human populations across the globe owing to the differences in their variations in risk allele frequencies.[25] This provides the impetus to perform region-specific genetic association tests to identify the disease-associated SNPs. A re-investigation is therefore warranted to correctly understand the role of *FEN1* gene in Polish individuals affected with FECD.

Although these *FEN1* polymorphisms are not genetically associated with FECD, researchers have associated c.4150G>T (rs4246215) with deregulated *FEN1* transcript expression in lung- and breast cancer tissues [23, 26]. Genetic association analysis in patients diagnosed with hepatocellular, esophageal, gastric, colorectal, and glial cell cancers have reported that the homozygous c.4150T allele limits cancer progression [27–29]. Lung tissues of c.4150TT homozygotes showed reduced DNA damage and *FEN1* transcripts as compared to c.4150GG. Researchers hypothesized that increased *FEN1* mRNA expression in lung [23] and gastrointestinal [28] cancers in c.4150TT homozygotes could be due to unsuccessful binding of miRNA. Through bioinformatics analysis, we identified that hsa-miR-1236-3p could be a potential regulator of *FEN1* transcription. Reporter assay and genotype-specific expression analysis of *FEN1* gene validated that hsa-miR-1236-3p specifically targets the major allele “G” at c.4150G>T SNP position. This report provides a better understanding of designing therapeutic targets to allow *FEN1* expression in affected tissues.

The current study disproves the genetic association of *FEN1* polymorphisms with FECD and cautions the readers to appropriately interpret the association results reported in Polish population. It also identifies a novel miRNA candidate that might target one of the 3’UTR SNP and regulate *FEN1* expression.
